# Case selection and causal inferences in qualitative comparative research

**DOI:** 10.1371/journal.pone.0219727

**Published:** 2019-07-24

**Authors:** Thomas Plümper, Vera E. Troeger, Eric Neumayer

**Affiliations:** 1 Department of Socioeconomics, Vienna University of Economics and Business, Vienna, Austria; 2 Department of Economics, University of Warwick, Coventry, United Kingdom; 3 Department of Geography and Environment, London School of Economics and Political Science, London, United Kingdom; University of California-Irvine, UNITED STATES

## Abstract

Traditionally, social scientists perceived causality as regularity. As a consequence, qualitative comparative case study research was regarded as unsuitable for drawing causal inferences since a few cases cannot establish regularity. The dominant perception of causality has changed, however. Nowadays, social scientists define and identify causality through the counterfactual effect of a treatment. This brings causal inference in qualitative comparative research back on the agenda since comparative case studies can identify counterfactual treatment effects. We argue that the validity of causal inferences from the comparative study of cases depends on the employed case-selection algorithm. We employ Monte Carlo techniques to demonstrate that different case-selection rules strongly differ in their ex ante reliability for making valid causal inferences and identify the most and the least reliable case selection rules.

## Introduction

We demonstrate that the validity of causal inferences based on the qualitative comparison of cases depends on the data-generating process and on the choice of case-selection algorithm. While the first factor is beyond the influence of scientists, researchers can freely choose the algorithm that determines the selection of cases. Of course, methodologists have long since been aware of the importance of case-selection for qualitative comparative research [1,2,3]. One can trace back systematic theoretical and methodological reasoning on case-selection to at least John Stuart Mill [4]. After all this time, one might expect that the optimal case-selection algorithms are known. Yet, this is only partially the case. We offer one of the first rigorous analyses of the relative performance of both simple and more complex case-selection rules under conditions of relevance to real world comparative research [5].

Specifically, we vary the size of the total set of cases from which specific cases are selected, we vary the degrees to which the causal factor of interest is correlated with confounding factors, and we vary the “signal-to-noise ratio”, that is, the (relative) strength of the effect of the causal factor of interest. Using a Monte Carlo design we compare the relative performance of 11 case-selection algorithms, partly following suggestions of qualitative methodologists and partly derived from common practice in comparative case analyses. The very best case-selection algorithm results in an estimated average effect that is almost a hundred times closer to the true effect than the worst algorithm. We also evaluate the conditions conducive to higher validity of causal inferences from qualitative comparative research. We find that the best selection algorithms exhibit relatively high ex ante reliability for making valid inferences if: a) the explanatory variable of interest exerts a strong influence on the dependent variable relative to random noise and confounding factors, b) the variable of interest is not too strongly correlated with confounding variables, and c) the dependent variable is not dichotomous. More importantly, while the best algorithms are still fairly reliable even in the presence of strong stochastic influences on the dependent variable and other complications, the worst algorithms are highly unreliable even if the conditions are met under which qualitative comparative research works best.

Our research contributes to both qualitative and quantitative methodological debates. Quantitative researchers assume that it is impossible to derive valid causal inferences from qualitative comparative research methods. However, we argue that this assumption is outdated because the concept of causality as regularity [6,4,7] has been superseded by the concept of causality as counterfactual effect [8,9,10]. In fact, the counterfactual concept of causation requires only a single case for causal inference if only it were possible to observe the counterfactual [11,12,13]. In the absence of directly observable counterfactual outcomes, the closest methodological equivalents according to the ‘identification school’ are randomization of treatment [14] and stratification of treatment and control group [15] through case-selection. It is this latter research strategy of rule- or model-based case-selection that demands a re-evaluation of qualitative comparative designs.

The logic of causal inference typically invoked by quantitative methodologists therefore also applies to qualitative comparative methods: if two or more cases are identical in all relevant dimensions but vary in the treatment, causal inference is internally valid. In addition, our research demonstrates that if these two cases are sampled so that the difference in the treatment is maximized the precision of the computed causal effect is large. We understand of course that these optimal conditions often do not exist and that selected cases vary in more dimensions than the treatment effect. Analyzing how different case-selection rules perform as a function of different conditions in which they must operate is exactly the purpose of our contribution.

As for the debate amongst qualitative methodologists, our results first and foremost speak to qualitative comparative researchers who, in the tradition of John Stuart Mill, draw inferences from the comparison of two sufficiently similar cases that vary in respect to the variable of interest (the ‘treatment’). Yet, the research design logic supported by our results also applies to scholars who compare a single case at two or more different points in time with a ‘treatment’ occurring in between the first and the last observation of the selected single case. These research designs are comparative in nature, and thus our findings that inferences are most likely to be valid if researchers maximize the variance of the variable of interest and minimize the variance of the confounding factors for selecting the case or cases they analyze over time also holds for a comparison of two different observations in time of a single case.

Yet, our research also contrasts with some of the acquired wisdom of qualitative methodologists. We agree that qualitative research, including the in-depth study of one or more cases and the comparative study of cases, can serve many other purposes and are, arguably, better suited for inductive purposes such as theory and concept development [16,17]. Qualitative research often seeks to generate ideas about the data-generating process so that little knowledge of the data-generating process can be assumed to exist prior to the case selection. Clearly, the logic of case selection for deductive causal inference research differs from the logic of case selection for inductive research. We therefore do not believe that our results can or indeed should be extended to inductive research. Importantly, however, many empirical qualitative researchers do make causal inferences and generalize their findings from the analyzed cases to a broader population. Our analysis enables those qualitative researchers who do wish to make causal inferences based on the comparative analysis of cases to understand how case-selection rules differ with respect to their *ex ante* reliability for detecting the direction and strength of a causal effect. Crucially, given limited knowledge about the data-generating process, we show that the relatively best-performing algorithms remain best-performing no matter what the underlying data-generating process (of those we have analyzed).

Qualitative researchers might struggle with a second aspect of our research design. Qualitative comparative researchers hardly ever estimate the strength of an effect and thus an analysis of effect strengths must seem irrelevant for them (but see [18]). Yet, we do not compute the effect strength from a comparison of two cases to tempt qualitative researchers to quantify effect strengths. We merely compute the effect strength and compare it to the assumed true effect size to have an indicator against which we can judge the *ex ante* reliability of selection algorithms. Computing the effect size is a tool, not the goal. Even if qualitative comparative researchers only intend to make inferences on the direction of a causal effect, they should agree that the expected deviation of an implied effect strength estimate from the truth–called root mean squared error by the quantitative tribe–is a good indicator for the relative *ex ante* reliability of case-selection algorithms: The larger this deviation, the more likely that even the inferred direction of an effect is wrong.

The paper is organized as follows: the next section shows that the now dominant modern concept of causality as counterfactual analysis implies that one can make causal inferences based on qualitative comparative analysis. One cannot make such inferences with certainty, however, and the validity of inferences will crucially depend on how cases are selected. We review what methodologists have advised on the selection of cases in qualitative comparative research in section 3. This informs our choice of selection algorithms that we subject to Monte Carlo analysis, though we also add some original algorithms to test whether and, if so, how much better they can perform. Section 4 describes these algorithms, the Monte Carlo design and how we evaluate the relative performance of the case-selection algorithms. Section 5 presents results from the Monte Carlo simulations.

## Causal inference and qualitative comparative research

Causality as regularity dominated the philosophy of science at least from Hume to Popper. Hume [5] argued that scientists cannot have knowledge of causality beyond observed regularities in associations of events. He therefore suggests inferring causality through a systematic comparison of situations in which the presumed causal factor is present or absent, or varies in strength. The concept of causality as regularity became the central element of Hempel and Oppenheim’s [19] deductive-nomological model of scientific explanation. Hempel was also the first to develop the concept further to include statistical inference [20]. In Popper’s conception of a non-degenerative research program [7], a single falsification effectively leads to the rejection of the tested hypothesis or, worse, the theory from which the hypothesis derives. The “regularity” perspective culminates in the definition of science as “unbroken, natural regularity” [21].

This “strict regularity” concept of causality had ambiguous implications for comparative social science qualitative researchers’ ability to make causal inferences. On the one hand, the analysis of a small number of cases cannot establish regularity. On the other hand, if, conversely, a single deviant case suffices to refute a causal claim or even a theory, as Popper believes, then strength in numbers does not exist [22,23,17]. The “strict regularity” perspective is dead, however, because a) not all regularities are causal (“correlation is not causation”) and b) causality can be probabilistic rather than deterministic and can thus exist without strict regularity.

Probabilistic causal mechanisms paved the way for an interpretation of regularity as statistical regularity. Yet, not even the brilliant idea of statistical inference saved the regularity concept of causality. If correlation is not causality, then high correlation does not imply causality either and low correlation and statistical insignificance may indicate low-probability causality and a lack of sufficient variation rather than the absence of causality. Eventually, this insight eliminated the support for the causality as regularity view.

Over the last three decades, the concept of causality as regularity was replaced by the counterfactual concept of causality, also called the potential outcomes framework. Its understanding of causality is tautological: causality exists if a cause exerts a causal effect on the outcome, and a cause exerts a causal effect on the outcome when the relation is causal. This tautology seems to be the main reason why scholars advancing the counterfactual perspective [9,10,24,25] focus on causal inference and the identification of causal effects rather than on causality itself [24].

According to the counterfactual concept of causality, causality is perfectly identified if one observes the outcome given treatment and the outcome given no treatment at the same time for the same person(s). Naturally, this is impossible. Hence, a counterfactual analysis starts with a ‘missing data’ problem and then immediately turns to ‘second-best’ options for inferring causality. If one cannot observe the potential or counterfactual outcome for any one single case, then one needs to resort to comparing the outcomes of different cases. This raises the challenge that either one must make sure that the cases compared are equal or sufficiently similar in all dimensions that matter or that one can render the influence of all potential confounders irrelevant. Otherwise, no causal effect has been ‘identified’.

The approach generally preferred by identification scholars–what they call the “gold standard”–aspires to render potential confounders irrelevant by randomizing treatment across a large number of cases in a controlled experiment (but see [25,26]). Though practically all actual experiments fall way short of the ideal of experimental designs, the randomization of treatments in a sample where *N* approaches infinity guarantees that the treatment will be uncorrelated with both observable and, crucially, unobservable confounders. Because of this lack of correlation with any potential confounder, any observable difference in outcomes between the two groups must be due to the treatment. If one assumes causal homogeneity among cases and assumes away that potential confounders might condition the effect of treatment, then ideal experiments will not only have identified a cause-effect relationship but will also allow the calculation of the unbiased effect size.

Clearly, from the experimentalist viewpoint, qualitative small-N comparative research is useless for causal inferences. In fact, so is everything else. Its diehard proponents explicitly argue that experiments are a necessary condition for causal inference. For example, Light, Singer, and Willett [27] claim that “to establish a causal link, you must conduct an *experiment* (…). Only experimental inquiries allow you to determine whether a treatment *causes* an outcome to change.” This claim wrongly assumes that identification is a necessary condition for causal inference, whereas in fact perfect identification is only a necessary condition for making causal inferences that are valid with certainty. The idea that one can only make causal inferences if scientists are certain about having identified a cause-effect relationship via experiments is absurd, however. If the claim was correct, scientists would not be able to infer that more education causes higher lifetime income, or that smoking causes lung cancer. For that matter, social scientists would not be able to explore much of interest. The quest for causal inference in the social sciences is not about certainty; it is about how to deal with uncertainty and how much uncertainty about the validity of inferences can be tolerated.

More importantly, making certainty a prerequisite for causal inference runs into a logical problem for the social sciences because experiments that social scientists are able to conduct do not generate inferences that are valid with certainty. Even ignoring causal heterogeneity and potential conditionalities [28], the confounding-factors problem can only be solved asymptotically, that is, by increasing the sample size to infinity. With a finite number of participants, randomization of treatment does not suffice to render treatment uncorrelated to unobserved confounders like mood, experience, knowledge, or intelligence, and often to even observed confounders like age, sex, income, or education. As a remedy, many experimenters control for observable differences in addition to randomizing treatment. Since it is impossible to control for all factors that influence human behavior, not least because some of them may be unobserved, the problem of confounders can be reduced but not eliminated by experiments. Yet, if experiments only increase the probability that causal inferences are correct, then the strict dichotomy between experiments and all other research methods that Light, Singer, and Willett make is unjustified.

The second approach to solving the “missing data” problem in the counterfactual concept of causality argues that causal effects are identified if cases can be selected so as to guarantee that all the relevant properties of the treatment group exactly match the properties of the control group [29,30,31]. Identification via selection or matching on the properties of the treatment and control groups requires perfect knowledge of all the factors that influence outcomes and also that one can match cases on these properties. As with experiments, falling short of this ideal will mean that a causal effect has not been identified with certainty, but does not render causal inference impossible. For experimentalists, matching is far inferior to experiments because they doubt one can know all the relevant properties (one can know the so-called data-generating process) and even if one could know these properties, one cannot measure all of these properties, some of which are unobservable, and thus one cannot match on them.

This second approach substitutes impossible counterfactual analyses with a possible analysis of cases that have been carefully selected to be homogeneous with respect to confounding variables. This strategy is obviously encouraging for causal inference based on case comparison. Nothing in this matching approach suggests that the validity of causal inferences depends on the number of cases. If cases are homogeneous, causal inferences based on small-N qualitative comparative methods become possible, and the validity of these causal inferences depends on the employed selection rule.

Qualitative comparative researchers have always made arguments that closely resemble matching [5]: if two cases are identical in all relevant dimensions but vary in the dimension of interest (the treatment), then it is possible to directly infer causality and to compute a causal effect size. This possibility does not imply that causal inference from qualitative comparative research is optimal or easy, however. Of course, there is the issue of knowing all relevant dimensions and finding at least two cases which are identical in all these dimensions. There are other difficulties, too: First, if causal processes are stochastic, as they are bound to be, then a single small-N comparative analysis, which cannot control for noise and random errors, will not reveal the truth but some random deviation from the truth. Matching cases in a quantitative analysis with large N therefore can be superior—though the greater difficulty of adequately matching a larger number of cases means that any positive effect on the validity of causal inferences from efficiency gains may be defeated by the negative effect due to problems in matching. Second, perfect homogeneity among cases on all confounding factors can only be achieved if researchers know the true data-generating process, which is unlikely to be the case even if qualitative researchers argue that their in-depth study of cases allow them to know much more about this process than quantitative researchers do [32,33]. In the absence of knowledge of the true data-generating process, qualitative comparative researchers should make sure that selected cases do not differ in respect to known strong confounding factors. The potential for bias grows with the strength of the potentially confounding factor (for which no controls have been included), and the size of the correlation between the variable of interest and the confounder.

## Case-selection and qualitative comparisons

Methodological advice on the selection of cases in qualitative research stands in a long tradition. John Stuart Mill in his *A System of Logic*, first published in 1843, proposed five methods meant to enable researchers to make causal inferences: the method of agreement, the method of difference, the double method of agreement and difference, the method of residues, and the method of concomitant variation [4]. Methodologists have questioned and criticized the usefulness and general applicability of Mill’s methods [34,35]. However, without doubt Mill’s proposals had a major and lasting impact on the development of the two most prominent modern methods, namely the “most similar” and “most different” comparative case-study designs [1,36,37].

Yet, as Seawright and Gerring [3] point out, these and other methods of case-selection are “poorly understood and often misapplied”. Qualitative researchers mean very different things when they invoke the same terms “most similar” or “most different” and usually the description of their research design is not precise enough to allow readers to assess exactly how cases have been chosen. Seawright and Gerring have therefore provided a formal definition and classification of these and other techniques of case-selection. They [3] suggest that “in its purest form” the “most similar” design chooses cases which appear to be identical on all controls (*z*) but different in the variable of interest (*x*). Lijphart [1] suggested what might be regarded a variant of this method that asks researchers to maximize “the ratio between the variance of the operative variables and the variance of the control variables”.

Naturally, the “most similar” technique is not easily applied because researchers find it difficult to match cases such that they are identical on all control variables. As Seawright and Gerring [3] concede: “Unfortunately, in most observational studies, the matching procedure described previously–known as exact matching–is impossible.” This impossibility has three sources: first, researchers usually do not know the true model and thus cannot match on all control variables. Second, even if known to affect the dependent variable, many variables remain unobserved. And third, even if all necessary pieces of information are available, two cases that are identical in all excluded variables may not exist.

Qualitative comparative researchers prefer the “most similar” technique, despite ambiguity in its definition and practical operationalization, to its main rival, the “most different” design. Seawright and Gerring [3] believe that this dominance of “most similar” over “most different” design is well justified. Defining the “most different” technique as choosing two cases that are identical in the outcome *y* and in the main variable of interest *x* but different in all control variables *z*, they argue that this technique does not generate much leverage. They criticize three points: first, the chosen cases never represent the entire population (if *x can* in fact vary in the population). Second, the lack of variation in *x* renders it impossible to identify causal effects. And third, elimination of rival hypotheses is impossible. As Gerring [38] formulates poignantly: “There is little point in pursuing cross-unit analysis if the units in question do not exhibit variation on the dimensions of theoretical interest and/or the researcher cannot manage to hold other, potentially confounding, factors constant.”

For comparative case studies, Seawright and Gerring also identify a third selection technique, which they label the “diverse” technique. It selects cases so as to “represent the full range of values characterizing X, Y, or some particular X/Y relationship” [3]. This definition is somewhat ambiguous and vague (“some particular relationship”), but one of the selection algorithms used below in our MC simulations captures the essence of this technique by simultaneously maximizing variation in *y* and *x*.

Perhaps surprisingly, King, Keohane and Verba’s [39] seminal contribution to qualitative research methodology discusses case-selection only from the perspective of unit homogeneity–broadly understood as constant effect assumption–and selection bias–defined as non-random selection of cases that are not statistically representative of the population. Selecting cases in a way that does not avoid selection bias negatively affects the generalizability of inferences. Random sampling from the population of cases would clearly avoid selection bias. Thus, given the prominence of selection bias in King et al.’s discussion of case-selection, the absence of random sampling in comparative research may appear surprising. But it is not. Random selection of cases leads to inferences which are correct on average when the number of conducted case studies approaches infinity, but the sampling deviation is extremely large. As a consequence, the reliability of single studies of randomly sampled cases remains low. The advice King and his co-authors give on case-selection, then, lends additional credibility to commonly chosen practices by qualitative comparative researchers, namely to avoid truncation of the dependent variable, to avoid selection on the dependent variable, while at the same time selecting according to the categories of the “key causal explanatory variable”. King et al. [39] also repeatedly claim that increasing the number of observations makes causal inferences more reliable. Qualitative methodologists have argued that this view, while correct in principle, does not do justice to qualitative research [40,41,42]. More importantly, they also suggest that the extent to which the logic of quantitative research can be superimposed on qualitative research designs has limits.

While there is a growing consensus on the importance of case-selection for comparative research, as yet very little overall agreement has emerged concerning the use of central terminology and the relative advantages of different case-selection rules. Scholars largely agree that random sampling is unsuitable for qualitative comparative research (but see [5]), but disagreement on sampling on the dependent variable, and the appropriate use of information from observable confounding factors persists. Our Monte Carlo analysis will shed light on this issue by exploring which selection algorithms are best suited under a variety of assumptions about the data-generating process.

## A Monte Carlo analysis of case-selection algorithms

In statistics, Monte Carlo experiments are employed to compare the performance of estimators. The term Monte Carlo experiments describes a broad set of techniques that randomly draw values from a probability distribution to add error to a predefined equation that serves as data-generating process. Since the truth is known, it is straightforward to compare the estimated or computed effects to the true effects. An estimator performs the better the smaller the average distance between the estimated effect and the truth. This average distance is usually called the root mean squared error.

Our Monte Carlo experiments follow this common practice in statistics and merely replace the estimators by a case-selection rule or algorithm. We compare selection rules commonly used in applied qualitative comparative research, as well as various simple permutations and extensions. Without loss of generality, we assume a data-generating process in which the dependent variable *y* is a linear function of a variable of interest *x*, a control variable *z* and an error term *ε*. Since we can interpret *z* as a vector of *k* control variables, we can generalize findings to analyses with multiple controls.

### Case-selection algorithms

Ignoring for the time being standard advice against sampling on the dependent variable, researchers might wish to maximize variation of *y*, maximize variation of *x*, minimize variation of *z* or some combination thereof. Employing addition and subtraction, the two most basic functions to aggregate information on more than one variable, leads to seven permutations of information from which to choose; together with random sampling this results in eight simple case-selection algorithms–see Table 1. The mathematical description of the selection algorithms, as shown in the last column of the table, relies on the set-up of the Monte Carlo analyses (described in the next section). In general, for each variable we generate Euclidean distance matrices, which are *N×N* matrices representing the difference or distance in a set of cases *i* and *j* forming the case-dyad *ij*. Starting from these distance matrices, we select two cases that follow a specific selection rule. For example, *max(x)* only considers the explanatory variable of interest, thereby ignoring the distance matrices for the dependent variable *y* and the control variable *z*. With *max(x)*, we select the two cases that represent the cell of the distance matrix with the largest distance value. We refrain from analyzing case-selection algorithms for qualitative research with more than two cases. Note, however, that all major results we show here carry over to selecting more than two cases based on a single algorithm. However, we do not yet know whether all our results carry over to analyses of more than two cases when researchers select cases based on different algorithms–a topic we will revisit in future research.

**Table 1:** Simple case-selection algorithms.

		sampling information			
	Name	max dist(y)	max dist(x)	min dist(z)	selection algorithm
1	random	no	no	no	random draw
2	max(y)	yes	no	no	max dist(y)
3	max(x)	no	yes	no	max dist(x)
4	min(z)	no	no	yes	min dist(z)
5	max(y)max(x)	yes	yes	no	max [dist(y)+dist(x)]
6	max(y)min(z)	yes	no	yes	max [dist(y)-dist(z)]
7	max(x)min(z)	no	yes	yes	max [dist(x)-dist(z)]
8	max(y)max(x)min(z)	yes	yes	yes	max [dist(y)+dist(x)-dist(z)]

Algorithm 1 does not use information (other than that a case belongs to the population), and samples cases randomly. We include this algorithm for completeness and because qualitative methodologists argue that random sampling–the gold standard for sampling in quantitative research–does not work well in small-N comparative research.

We incorporate the second algorithm–pure sampling on the dependent variable without regard to variation of either *x* or *z*–for the same completeness reason. Echoing Geddes [43], many scholars have argued that sampling on the dependent variable biases the results [39,44,45]. Geddes demonstrates that “selecting on the dependent variable” lies at the core of invalid results generated from qualitative comparative research in fields as diverse as economic development, social revolution, and inflation.

But does Geddes’s compelling critique of sampling on the dependent variable imply that applied researchers should entirely ignore information on the dependent variable when they also use information on the variable of interest or the confounding factors? Algorithms 5, 6, and 8 help us to explore this question. These rules include selection on the dependent variable in addition to selection on *x* and/or *z*. Theoretically, these algorithms should perform better than the algorithm 2, but we are more interested in analyzing how these biased algorithms perform in comparison to their counterparts, namely algorithms 3, 4, and 7, which, respectively, maximize variation of *x*, minimize variation of *z*, and simultaneously maximize variation of *x* and minimize variation of *z*, just as algorithms 5, 6 and 8 do, but this time without regard to variation of *y*.

Theoretically, one would expect algorithm 7 to outperform algorithms 3 and 4. Qualitative methodlogists such as Gerring and Seawright and Gerring [17,3] expect this outcome and we concur. Using more information must be preferable to using less information when it comes to sampling. This does not imply, however, that algorithm 7 necessarily offers the optimal selection rule for qualitative comparative research. Since information from at least two different variables has to be aggregated, researchers have at their disposal multiple possible algorithms that all aggregate information in different ways. For example, in addition to the simple unweighted sum (or difference) that we assume in Table 1, one can aggregate by multiplying or dividing the distances, and one can also weight the individual components.

Lijphart [1] has suggested an alternative function for aggregation, namely maximizing the ratio of the variance in *x* and *z*: *max[dist(x)/dist(z)]*. We include Lijphart’s suggestion as our algorithm 9 even though it suffers from a simple problem which reduces its usefulness: when the variance of the control variable *z* is smaller than 1.0, the variance of what Lijphart calls the operative variable *x* becomes increasingly unimportant for case-selection (unless of course the variation of the control variables is very similar across different pairs of cases). We solve this problem by also including in the competition an augmented version of Lijphart’s suggestion. This algorithm 10 adds one to the denominator of the algorithm proposed by Lijphart: *max[dist(x)/(1+dist(z))]*. Observe that adding one to the denominator prevents the algorithm from converging to *min[dist(z)]* when *dist(z)* becomes small. Finally, we add a variance-weighted version of algorithm 7 as our final algorithm 11 to check whether weighting improves on the simple algorithms. Table 2 summarizes the additional analyzed algorithms that aggregate information using more complicated functions.

**Table 2:** Case-selection algorithms with more complicated functions for aggregating information from more than one variable.

		sampling information			
	Name	max dist(y)	max dist(x)	min dist(z)	selection algorithm
9	lijphart	no	yes	yes	max [dist(x)/dist(z)]
10	augmented lijphart	no	yes	yes	max [dist(x)/(1+dist(z))]
11	weighted max(x)min(z)	no	yes	yes	max $\left[\frac{\mathrm{dist}(x)}{\mathrm{maxdist}(x)}-\frac{\mathrm{dist}(z)}{\mathrm{maxdist}(z)}\right]$

Note that thus far we have given the selection algorithms formal and technical labels, avoiding terminology of case-selection rules commonly used in the literature. Nevertheless, there are connections between some of the above algorithms and the terminology commonly used in the literature. For example, algorithms 2, 3 and 5 are variants of selection rules described by Seawright and Gerring [3] as “diverse” case-selection rules. Algorithms 2, 5, 6, and 8 all use information on variation of the dependent variable and are thus variants of selection on the dependent variable. More importantly, algorithms 4 and 7 as well as algorithms 9 to 11 seem to be variants of the most similar design. However, we do not call any of these algorithms “selection on the dependent variable” or “most similar”. The reason is that, as discussed above, there is a lack of consensus on terminology and different scholars prefer different labels and often mean different things when they invoke rules such as “sampling on the dependent variable” or “most similar”.

### The Monte Carlo design

The use of Monte Carlo techniques may appear to be strange to qualitative researchers. However, Monte Carlo simulations are perfectly suited for the purpose of exploring the *ex ante* reliability of case-selection algorithms. As we have explained above, Monte Carlo simulations provide insights into the expected accuracy of inferences given certain pre-defined properties of the data-generating process. While they are commonly used to compare estimators, one can equally use them to compare the performance of different sampling rules.

Monte Carlo simulations allow us to systematically change the data-generating process, and to explore the comparative advantages of different selection algorithms depending on the assumptions we make about the data-generating process. Possible systematic changes include variation in the assumed level of correlation between explanatory variables, the relative importance of uncertainty, the level of measurement error, and so on. Unsystematic changes are modelled by repeated random draws of the error term.

Specifically, we define various data-generating processes from which we draw a number of random samples, and then select two cases from each sample according to a specific algorithm, as defined above. As a consequence of the unaccounted error process, the computed effects from the various Monte Carlo simulations will deviate somewhat from the truth. Yet, since we confront all selection algorithms to the same set of data-generating processes, including the same error processes, performance differences must result from the algorithms themselves. These differences occur because different algorithms will select different pairs of cases *i* and *j*, and, as a consequence, the computed effect and the distance of this effect from the true effect differ. Our analysis explores to what extent a comparison of two cases allows researchers to estimate the effect that one explanatory variable, called *x*, exerts on a dependent variable, called *y*. We assume that this dependent variable *y* is a function of *x*, a single control variable *z*, which is observed, and some error term *ε*: *y_i_* = *βx_i_*+*γz_i_*+*ε_i_*, where *β, γ* represent coefficients and *ε* is an iid error process. Obviously, as var(*ε*) approaches zero, the data-generating process becomes increasingly deterministic. We follow the convention of quantitative methodology and assume that the error term is randomly drawn from a standard normal distribution. Note, however, that since we are not interested in asymptotic properties of case-selection algorithms, we could as well draw the error term from different distributions. This would have no consequence other than adding systematic bias to all algorithms alike. The process resembles what Gerring and McDermott [46] call a “spatial comparison” (a comparison across *n* observations), but our conclusions equally apply to “longitudinal” (a comparison across *t* periods) and “dynamic comparisons” (a comparison across *n*·*t* observations). We conducted simulations with both a continuous and a binary dependent variable. We report results for the continuous variable in detail in the next section and briefly summarize the results for the binary dependent variable with full results reported in the appendices.

There are different ways to think about the error term. First, usually scientists implicitly assume that the world is not perfectly determined and they allow for multiple equilibria which depend on random constellations or the free will of actors. In this respect, the error term accounts for the existence of behavioral randomness. Second, virtually all social scientists acknowledge the existence of systematic and unsystematic measurement error. The error term can be perceived as accounting for information that is partly uncertain. And third, the error term can be interpreted as model uncertainty–that is, as unobserved omitted variables also exerting an influence on the dependent variable. Only if randomness and free will, measurement error, and model uncertainty did not exist, would the inclusion of an error term make no sense.

We always draw *x* and *z* from a normal distribution, but, of course, alternative assumptions are possible. Given the low number of observations, it comes without loss in generality that we draw *ε* from a normal distribution with mean zero and standard deviation of 1.5; and, unless otherwise stated, all true coefficients take the value of 1.0; the standard deviation of variables is 1.0; correlations are 0.0; and the number of observations N, representing the size of the sample from which researchers can select cases, equals 100.

### Evaluating the results from the Monte Carlo simulations

We compare the reliability of inference on effect strength. Specifically, the effect size of *x* on *y* from a comparative case study with two cases equals

$$\hat{\beta }\left(x\right)=\frac{y_{i}-y_{j}}{x_{i}-x_{j}},$$ (1)

where subscripts [*i*,*j*] represent the two selected cases. We take the root mean squared error (RMSE) as our measure for the reliability of causal inference as it reacts to both bias and inefficiency. The RMSE is defined as

$$RMSE=\sqrt{\frac{{\sum \left(\hat{\beta }-\beta_{true}\right)}^{2}}{N}}=\sqrt{Var\left(\hat{\beta }\right)+\left[Bias{\left(\hat{\beta },\beta_{true}\right)}^{2}\right]}.$$ (2)

This criterion not only incorporates bias (the average deviation of the computed effect from the true effect), but also accounts for inefficiency, which is a measure of the sampling variation of the computed effect that reflects the influence of random noise on the computed effect. Qualitative researchers cannot appropriately control for the influence of noise on estimates. The best they can do to account for randomness is to choose a case-selection algorithm that responds less than others to noise. Naturally, these are case-selection algorithms that make best use of information. In quantitative research, the property characterizing the best use of information is called *efficiency*, and we see no reason to deviate from this terminology.

## Results from the Monte Carlo analysis of case-selection algorithms

We conduct three sets of MC simulations, in which we vary the parameters of the data-generating process, and evaluate the effect of this variation on the precision with which the algorithms approach the true coefficients together with the efficiency of the estimation. In each type of analysis we draw 1,000 samples from the underlying data-generating process. In the first set of simulations, we change the number of observations from which the two cases are chosen (*i* = 1,…N), thereby varying the size of the sample, i.e., the total number of cases from which researchers can select two cases. In the second set of simulations, we vary the correlation between *x* and *z*–that is, the correlation between the variable of interest and the confounding factor. In the final set of simulations, we vary the variance of *x* and thus the effect size or explanatory power of *x* relative to the effect size of the confounding factor *z*.

Analyzing the impact of varying the number of analyzed cases on the validity of inferences in qualitative comparative research may seem strange at first glance. After all, qualitative researchers usually study a fairly limited number of cases. In fact, in our Monte Carlo analyses we generate effects by looking at a single pair of cases selected by each of the case-selection algorithms. So why should the number of cases from which we select the two cases matter? The reason is that if qualitative researchers can choose from a larger number of cases about which they have theoretically relevant information, they will be able to select a better pair of cases given the chosen algorithm. The more information researchers have before they select cases, the more reliable their inferences should thus become. In other words, N does not represent the number of cases analyzed, but the number of the total set of cases from which the analyzed cases are chosen.

By varying the correlation between *x* and the control variable *z* we can analyze the impact of confounding factors on the performance of the case-selection algorithms. With increasing correlation, inferences should become less reliable. Thereby, we look at the effect of potential model misspecification on the validity of inference in qualitative comparative research. While quantitative researchers can eliminate the potential for bias from correlated control variables by including these on the right-hand-side of the regression model, qualitative researchers have to use appropriate case-selection rules to reduce the potential for bias.

Finally, in varying the standard deviation of *x* we analyze the impact of varying the strength of the effect of the variable of interest on the dependent variable. The larger this relative effect size of the variable of interest, the more reliable causal inferences should become. The smaller the effect of the variable of interest *x* on *y* in comparison to the effect on *y* of the control or confounding variables *z*, the harder it is to identify the effect correctly, and the less valid the inferences become–especially when the researcher does not know the true specification of the model.

Table 3 reports the Monte Carlo results obtained when we only vary the size of the sample from which we draw the two cases we compare. In this set of simulations, we do not allow for systematic correlation between the variable of interest *x* and the confounding factor *z*. The deviations of computed effects from the true effect occur because of “normal” sampling error, and how efficiently the algorithm deals with the available information.

**Table 3:** Monte Carlo results from varying ‘population size’.

	Algorithm	N = 20	N = 40	N = 60	N = 80	N = 100
1	random	9.137	13.846	6.008	6.860	17.349
2	max(y)	65.096	16.481	55.532	7.604	12.787
3	max(x)	0.575	0.488	0.447	0.429	0.411
4	min(z)	23.399	10.234	40.154	18.113	6.929
5	max(y)max(x)	3.213	7.608	35.725	1.935	2.047
6	max(y)min(z)	13.072	5.915	14.028	7.241	9.997
7	max(x)min(z)	0.522	0.438	0.419	0.387	0.360
8	max(y)max(x)min(z)	2.925	2.014	1.704	1.505	1.563
9	lijphart	1.754	1.544	1.400	1.548	1.416
10	augmented lijphart	0.536	0.479	0.442	0.407	0.389
11	weighted max(x)min(z)	0.521	0.442	0.417	0.388	0.359

Note: corr(x,z) = 0, SD(x) = 1

The table displays the root mean squared error. Smaller numbers indicate higher reliability.

Observe, first, that of the basic case-selection algorithms, *max(x)min(z)* performs up to 100 times better with respect to the average deviation from the true effect (the root mean squared error) than the poorest-performing competitors, namely *random*, which draws two cases randomly from the sample, and *max(y)*, which purely selects on the dependent variable. The drawback from selecting on the dependent variable declines if researchers additionally take into account variation of *x* and/or variation of *z*, but these algorithms 5, 6, and 8 are typically inferior to their counterparts 3, 4, and 7, which ignore variation of the dependent variable. Accordingly, selection on the dependent variable not only leads to unreliable inferences that are likely to be wrong, it also makes other selection algorithms less reliable. Hence, researchers should not pay attention to variation in the dependent variable *y* when they select cases. By selecting cases on the variable of interest *x* while at the same time controlling for the influence of confounding factors, researchers are likely to choose cases which vary in their outcome if *x* indeed exerts an effect on *y*.

Maximizing variation of *x* while at the same time minimizing variation of *z* appears optimal. Algorithm 7 uses subtraction as a basic function for aggregating information from more than one variable. Would using a more complicated function dramatically improve the performance of case-selection? The results reported in Table 3 show that, at least for this set of simulations, this is not the case. Algorithm 7 performs roughly 10 percent better than the augmented version of Lijphart’s proposal (*augmented lijphart*), and while algorithm 11, the variance-weighted version of algorithm 7, is very slightly superior, not much separates the performance of the two.

Another interesting finding from Table 3 is that only four algorithms become systematically more reliable when the population size from which we draw two cases increases. These four algorithms are: *max(x)*, *max(x)min(z)* and its weighted variant, *weighted max(x)min(z)*, as well as *augmented lijphart*. Algorithms need to have a certain quality to generate, in expectation, improvements in the validity of causal inferences when the population size becomes larger. Random selection, for example, only improves on average if the increase in population size leads to relatively more “onliers” than “outliers”. This may be the case, but there is no guarantee. When researchers use relatively reliable case-selection algorithms, however, an increase in the size of the sample, on which information is available, improves causal inferences unless one adds extreme outliers to the sample. Inferences become more reliable if cases are selected from a larger sample of cases for which researchers have sufficient information. We are not making any normative claim about enlarging the population size, because the improvements of enlarging the population from which cases are selected has to be discounted by the deteriorations caused by an increase in case heterogeneity caused by an enlarged sample.

The results from Table 3 support King, Keohane and Verba’s [39] arguments against both random selection and sampling on the dependent variable. At first sight, our results seem to differ from Herron and Quinn’s [5] finding that “simple random sampling outperforms most methods of case selection” even when the number of analyzed cases “is as small as 5 or 7”. However, our results are consistent with Herron and Quinn’s finding that random sampling is not reliable when the number of cases is two. In fact, the number of cases required to make random sampling a viable strategy depends on the heterogeneity of cases and the signal-to-noise ratio of the causal effect of interest: the more homogeneous and stronger the effect researchers are interested in, the better the performance of random selection of cases and the lower the number of cases for sufficiently reliable inferences.

In Table 4, we report the results of Monte Carlo simulations from varying the correlation between the variable of interest *x* and the confounding factor *z*.

**Table 4:** Monte Carlo results from varying the correlation between the variable of interest *x* and the confounding factor *z*.

	Algorithm	corr = -0.9	corr = -0.7	corr = -0.3	corr = 0	corr = 0.3	corr = 0.7	corr = 0.9
1	random	10.849	6.188	11.002	7.301	10.535	5.783	7.420
2	max(y)	52.987	64.685	13.840	7.154	4.215	2.883	2.379
3	max(x)	0.891	0.733	0.465	0.401	0.472	0.734	0.930
4	min(z)	20.962	8.325	5.717	5.653	8.742	10.358	36.662
5	max(y)max(x)	2.801	2.777	2.177	1.944	1.929	1.799	1.822
6	max(y)min(z)	61.050	19.741	6.171	4.685	9.976	11.658	4.980
7	max(x)min(z)	0.741	0.486	0.369	0.364	0.383	0.475	0.711
8	max(y)max(x)min(z)	10.010	2.787	1.591	1.520	1.666	1.981	2.159
9	lijphart	3.426	2.202	1.671	1.575	1.505	2.072	3.778
10	augmented lijphart	0.869	0.551	0.397	0.372	0.411	0.543	0.829
11	weighted max(x)min(z)	0.736	0.481	0.369	0.363	0.383	0.472	0.701

Note: SD(x) = 1.0, N = 100, SD(z) = 1.0, Varying Correlation (x,z). The table displays the root mean squared error. Smaller numbers indicate higher reliability.

Note that all substantive results from Table 3 remain valid if we allow for correlation between the variable of interest and the confounding factor. In particular, algorithm 11, which weights the individual components of the best-performing simple case-selection algorithm 7, performs only very slightly better; while the performance gap between simple algorithm *max(x)min(z)*, based on subtraction, and the augmented Lijphart algorithm (*augmented lijphart*), which uses the ratio as aggregation function, increases only marginally. Table 4 also demonstrates that correlation between the variable of interest and confounding factors renders causal inferences from qualitative comparative research less reliable. Over all simulations and algorithms, the RMSE increases by at least 100 percent when the correlation between *x* and *z* increases from 0.0 to either -0.9 or +0.9.

Finally, we examine how algorithms respond to variation in the strength of the effect of the variable of interest. In this final set of simulations for which results are reported in Table 5 we vary the standard deviation of the explanatory factor *x*; a small standard deviation indicates a small effect of *x* on *y* relative to the effect exerted from *z* on *y*. The results show that the performance of all case-selection algorithms suffers from a low “signal-to-noise” ratio. As one would expect, the smaller the effect of the variable of interest *x* on *y* relative to the effect of *z* on *y*, the less reliable the causal inferences from comparative case study research becomes. Yet, we find that the algorithms which performed best in the previous two sets of simulations also turn out to be least vulnerable to a small effect of the variable of interest. Accordingly, while inferences do become more unreliable when the effect of the variable of interest becomes small relative to the total variation of the dependent variable, comparative case studies are not simply confined to analyzing the main determinant of the phenomenon of interest if one of the top performing case-selection algorithms are used. As in the previous sets of simulations, we find that little is gained by employing more complicated functions for aggregating information from more than one variable as, for example, the ratio (*augmented lijphart*) or weighting by the variance of *x* and *z* (*weighted max(x)min(z)*). Sticking to the most basic aggregation function has little cost, if any.

**Table 5:** Monte Carlo results from varying the strength of the effect of the variable of interest.

	algorithm	SD(x) = 0.3	SD(x) = 0.7	SD(x) = 1.0	SD(x) = 1.5	SD(x) = 2.0
1	random	20.796	13.926	7.301	4.701	12.342
2	max(y)	105.183	22.097	7.154	2.706	0.969
3	max(x)	1.390	0.597	0.401	0.274	0.200
4	min(z)	41.889	13.112	5.653	8.377	3.024
5	max(y)max(x)	56.402	6.168	1.944	0.803	0.456
6	max(y)min(z)	125.917	68.193	4.685	1.671	0.738
7	max(x)min(z)	1.291	0.521	0.364	0.236	0.177
8	max(y)max(x)min(z)	95.349	3.862	1.520	0.654	0.388
9	lijphart	4.842	2.153	1.575	0.956	0.730
10	augmented lijphart	1.293	0.542	0.372	0.259	0.197
11	weighted max(x)min(z)	1.242	0.522	0.363	0.233	0.177

Note: corr(x,z) = 0.0, N = 100, SD(z) = 1.0, Varying SD(x)

The table displays the root mean squared error. Smaller numbers indicate higher reliability.

We now briefly report results from additional Monte Carlo simulations which we show in full in the appendix to the paper (S1 File). First, weighting *x* and *z* by their respective sample range becomes more important when the data-generating process includes correlation between *x* and *z* and the effect of *x* on *y* is relatively small (see Table A in S1 File). In this case, weighting both the variation of *x* and *z* before using the *max(x)min(z)* selection rule for identifying two cases slightly increases the reliability of causal inferences.

Second, we also conducted the full range of Monte Carlo simulations with a dichotomous dependent variable (see Tables B- E in S1 File). We find that the algorithms that perform best with a continuous dependent variable also dominate with respect to reliability when we analyze dichotomous dependent variables. Yet, causal inferences from qualitative comparative case study research become far less reliable when the dependent variable is dichotomous for all selection algorithms compared to the case of a continuous dependent variable. The root mean squared error roughly doubles for the better-performing algorithms. As a consequence, causal inferences with a binary dependent variable and an additional complication (either a non-trivial correlation between *x* and *z* or a relatively small effect of *x* on *y*) are not reliable. Accordingly, qualitative researchers should not throw away variation by dichotomizing their dependent variable. Where the dependent variable is dichotomous, qualitative comparative research is confined to what most qualitative researchers actually do in these situations: trying to identify strong and deterministic relationships or necessary conditions [47,48]. In both cases, the strong deterministic effect of *x* on *y* compensates for the low level of information in the data.

## Conclusion

Case-selection rules employed in qualitative research resemble ‘matching’ algorithms developed by identification scholars in quantitative research and thus can be employed to derive causal inferences. They also share their most important shortcoming: the extent to which causal inferences from selected samples are valid is partly determined by the extent of knowledge of the data-generating process. The more is known about the “true model”, the better researchers can select cases to maximize the *ex ante* reliability of their causal inferences.

Our major contribution has been to guide qualitative comparative researchers on what are the selection rules with the highest *ex ante* reliability for the purpose of making causal inferences under a range of conditions regarding the underlying data-generating process. The validity of causal inferences from qualitative comparative research will necessarily always be uncertain but following our guidance will allow qualitative comparative researchers to maximize the imperfect validity of their inferences.

Qualitative comparative researchers can take away six important concrete lessons from our Monte Carlo simulations: First, ceteris paribus, selecting cases from a larger set of potential cases gives more reliable results. Qualitative researchers often deal with extremely small samples. Sometimes nothing can be done to increase sample size, but where there are no binding constraints it can well be worth the effort expanding the sample from which cases can be selected. Second, for all the better-performing selection algorithms, it holds that ignoring information on the dependent variable for the purpose of selecting cases makes inferences much more reliable. Tempting though it may seem, qualitative comparative researchers should not select on the dependent variable at all. Third, selecting cases based on both the variable of interest and confounding factors improves the *ex ante* reliability of causal inferences in comparison to selection algorithms that consider just the variable of interest or just confounding factors–even if this means that one no longer chooses the cases that match most closely on confounding factors. These algorithms are relatively best-performing, no matter what the underlying data-generating process (of those we have analyzed). This is a crucial lesson because qualitative comparative researchers might not have much knowledge about the kind of data-generating process they are dealing with. Fourth, correlation between the variable of interest and confounding factors renders the selection algorithms less reliable. The same holds if the analyzed effect is weak. This reinforces existing views that qualitative case comparison is most suitable for studying strong and deterministic causal relationships [47,48]. Fifth, the reliability of case-selection rules depends on the variation in the dependent variable scholars can analyze. Accordingly, unless there are very strong over-riding theoretical or conceptual reasons, throwing away information by dichotomizing the dependent variable is a bad idea. A continuous dependent variable allows for more valid inferences; a dichotomous dependent variable should only be used if there is no alternative. Sixth, employing basic functions for aggregating information from more than one variable (such as maximizing the difference between variation of *x* and variation of *z*) does not reduce by much the *ex ante* reliability of case-selection compared to more complicated aggregation functions (such as maximizing the ratio or the variance-weighted difference). The only exceptions occur if *x* and *z* are highly correlated and the effect of *x* on *y* is relatively small compared to the effect of *z* on *y*. As a general rule, one does not lose much by opting for the most basic aggregation function.

In conclusion, our Monte Carlo study is broadly consistent with the views of qualitative methodologists. After all, the best- or nearly best-performing algorithms in our analysis of alternative selection algorithms appear to be variants of the most similar design, which in turn draws on Przeworski and Teune’s [35] and Lijphart’s [49] suggestions for case-selection. However, we are the first to provide systematic evidence that upholds existing recommendations in the presence of stochastic error processes. In addition, we demonstrated that simple functions for linking variation of the explanatory variable with variation of the confounding variables perform relatively well in general. There is little reason to resort to more advanced functions unless the explanatory variable has a weak effect and is strongly correlated with the confounding variables. One important area for further analysis comes from settings in which comparative qualitative researchers assess claims about two or more causal factors interacting with each other.

## Supporting information

S1 File Supporting information. (ZIP)
