# Supplementary material for: Case selection and causal inferences in qualitative comparative research
Source: PLoS One. 2019 Jul 24;14(7):e0219727. doi: 10.1371/journal.pone.0219727 (PMC6655636; doi:10.1371/journal.pone.0219727)
Supplement: S1 File — (ZIP) [file pone.0219727.s001.zip › Table A.docx]

Table A: MC Results Continuous Outcome SD(x)=0.3, N=100, SD(z)=1.0, Varying Correlation (x,z)

|  | Algorithm | corr=-0.9 | corr=-0.7 | corr=-0.3 | corr=0 | corr=0.3 | corr=0.7 | corr=0.9 |
| --- | --- | --- | --- | --- | --- | --- | --- | --- |
| 1 | random | 20.411 | 26.946 | 25.505 | 20.796 | 43.789 | 49.871 | 31.853 |
| 2 | max(y) | 57.993 | 99.264 | 270.517 | 105.183 | 136.001 | 21.068 | 16.749 |
| 3 | max(x) | 3.021 | 2.402 | 1.572 | 1.390 | 1.610 | 2.524 | 2.908 |
| 4 | min(z) | 69.408 | 27.372 | 32.238 | 41.889 | 20.927 | 373.118 | 68.821 |
| 5 | max(y)max(x) | 21.081 | 49.380 | 83.554 | 56.402 | 32.309 | 13.379 | 11.375 |
| 6 | max(y)min(z) | 342.961 | 2005.662 | 105.559 | 125.917 | 197.299 | 86.462 | 99.143 |
| 7 | max(x)min(z) | 3.015 | 1.862 | 1.380 | 1.291 | 1.343 | 1.819 | 3.100 |
| 8 | max(y)max(x)min(z) | 146.883 | 80.848 | 43.529 | 95.349 | 122.412 | 35.729 | 40.032 |
| 9 | lijphart | 11.206 | 7.000 | 4.714 | 4.842 | 5.319 | 7.246 | 11.404 |
| 10 | augmented lijphart | 2.773 | 1.871 | 1.377 | 1.293 | 1.340 | 1.800 | 2.851 |
| 11 | weighted max(x)min(z) | 2.319 | 1.668 | 1.277 | 1.242 | 1.207 | 1.611 | 2.350 |

Note: The table displays the root mean squared error. Smaller numbers indicate higher reliability.
