# Supplementary material for: Case selection and causal inferences in qualitative comparative research
Source: PLoS One. 2019 Jul 24;14(7):e0219727. doi: 10.1371/journal.pone.0219727 (PMC6655636; doi:10.1371/journal.pone.0219727)
Supplement: S1 File — (ZIP) [file pone.0219727.s001.zip › Table B.docx]

Table B: MC Results Binary Outcome SD(x)=1.0, SD(z)=1.0, corr(x,z)=0, Varying Population Size N

|  | Algorithm | N=20 | N=40 | N=60 | N=80 | N=100 |
| --- | --- | --- | --- | --- | --- | --- |
| 1 | random | 3.625 | 2.637 | 1.906 | 2.927 | 1.638 |
| 2 | max(y) | 3.044 | 3.130 | 3.361 | 10.140 | 7.093 |
| 3 | max(x) | 0.818 | 0.825 | 0.829 | 0.836 | 0.831 |
| 4 | min(z) | 3.039 | 1.745 | 6.143 | 4.306 | 2.881 |
| 5 | max(y)max(x) | 0.767 | 0.785 | 0.794 | 0.804 | 0.803 |
| 6 | max(y)min(z) | 3.914 | 3.184 | 7.053 | 5.224 | 5.022 |
| 7 | max(x)min(z) | 0.813 | 0.805 | 0.823 | 0.824 | 0.826 |
| 8 | max(y)max(x)min(z) | 0.732 | 0.756 | 0.778 | 0.783 | 0.787 |
| 9 | lijphart | 0.927 | 0.945 | 0.909 | 0.908 | 0.911 |
| 10 | augmented lijphart | 0.811 | 0.803 | 0.819 | 0.823 | 0.819 |
| 11 | weighted max(x)min(z) | 0.812 | 0.807 | 0.823 | 0.823 | 0.825 |

Note: The table displays the root mean squared error. Smaller numbers indicate higher reliability.
