# Supplementary material for: Case selection and causal inferences in qualitative comparative research
Source: PLoS One. 2019 Jul 24;14(7):e0219727. doi: 10.1371/journal.pone.0219727 (PMC6655636; doi:10.1371/journal.pone.0219727)
Supplement: S1 File — (ZIP) [file pone.0219727.s001.zip › Table C.docx]

Table C: MC Results Binary Outcome SD(x)=1.0, N=100, SD(z)=1.0, Varying Correlation (x,z)

|  | Algorithm | corr=-0.9 | corr=-0.7 | corr=-0.3 | corr=0 | corr=0.3 | corr=0.7 | corr=0.9 |
| --- | --- | --- | --- | --- | --- | --- | --- | --- |
| 1 | random | 8.114 | 2.672 | 4.221 | 2.600 | 1.754 | 1.308 | 1.983 |
| 2 | max(y) | 4.651 | 6.662 | 3.687 | 6.796 | 2.787 | 3.281 | 7.919 |
| 3 | max(x) | 0.974 | 0.924 | 0.861 | 0.833 | 0.813 | 0.802 | 0.799 |
| 4 | min(z) | 4.779 | 3.220 | 1.789 | 1.921 | 2.073 | 1.742 | 5.435 |
| 5 | max(y)max(x) | 0.967 | 0.894 | 0.830 | 0.810 | 0.798 | 0.799 | 0.798 |
| 6 | max(y)min(z) | 10.050 | 4.631 | 2.729 | 4.534 | 3.888 | 4.997 | 9.693 |
| 7 | max(x)min(z) | 0.806 | 0.802 | 0.820 | 0.830 | 0.823 | 0.818 | 0.800 |
| 8 | max(y)max(x)min(z) | 0.729 | 0.755 | 0.784 | 0.791 | 0.778 | 0.742 | 0.681 |
| 9 | lijphart | 1.319 | 1.025 | 0.942 | 0.928 | 0.875 | 0.965 | 1.309 |
| 10 | augmented lijphart | 0.768 | 0.785 | 0.816 | 0.823 | 0.821 | 0.818 | 0.801 |
| 11 | weighted max(x)min(z) | 0.801 | 0.804 | 0.820 | 0.830 | 0.824 | 0.816 | 0.795 |

Note: The table displays the root mean squared error. Smaller numbers indicate higher reliability.
