# Supplementary material for: Case selection and causal inferences in qualitative comparative research
Source: PLoS One. 2019 Jul 24;14(7):e0219727. doi: 10.1371/journal.pone.0219727 (PMC6655636; doi:10.1371/journal.pone.0219727)
Supplement: S1 File — (ZIP) [file pone.0219727.s001.zip › Table D.docx]

Table D: MC Results Binary Outcome SD(x)=1.5, N=100, SD(z)=1.0, Varying Correlation (x,z)

|  | Algorithm | corr=-0.9 | corr=-0.7 | corr=-0.3 | corr=0 | corr=0.3 | corr=0.7 | corr=0.9 |
| --- | --- | --- | --- | --- | --- | --- | --- | --- |
| 1 | random | 1.411 | 1.445 | 1.794 | 1.420 | 2.306 | 1.835 | 1.224 |
| 2 | max(y) | 4.220 | 5.267 | 3.399 | 4.789 | 4.503 | 3.064 | 1.634 |
| 3 | max(x) | 0.911 | 0.901 | 0.881 | 0.871 | 0.866 | 0.865 | 0.865 |
| 4 | min(z) | 2.891 | 1.568 | 1.556 | 1.273 | 1.441 | 4.080 | 2.394 |
| 5 | max(y)max(x) | 0.890 | 0.878 | 0.869 | 0.865 | 0.864 | 0.865 | 0.865 |
| 6 | max(y)min(z) | 5.553 | 15.612 | 3.498 | 1.863 | 1.623 | 5.266 | 6.293 |
| 7 | max(x)min(z) | 0.871 | 0.851 | 0.865 | 0.867 | 0.865 | 0.854 | 0.843 |
| 8 | max(y)max(x)min(z) | 0.842 | 0.837 | 0.856 | 0.859 | 0.855 | 0.836 | 0.831 |
| 9 | lijphart | 0.997 | 0.858 | 0.858 | 0.843 | 0.862 | 0.929 | 1.018 |
| 10 | augmented lijphart | 0.780 | 0.823 | 0.852 | 0.859 | 0.858 | 0.848 | 0.839 |
| 11 | weighted max(x)min(z) | 0.812 | 0.834 | 0.861 | 0.865 | 0.864 | 0.854 | 0.833 |

Note: The table displays the root mean squared error. Smaller numbers indicate higher reliability.
