# Supplementary material for: Case selection and causal inferences in qualitative comparative research
Source: PLoS One. 2019 Jul 24;14(7):e0219727. doi: 10.1371/journal.pone.0219727 (PMC6655636; doi:10.1371/journal.pone.0219727)
Supplement: S1 File — (ZIP) [file pone.0219727.s001.zip › Table E.docx]

Table E: MC Results Binary Outcome SD(x)=0.3, N=100, SD(z)=1.0, Varying Correlation (x,z)

|  | Algorithm | corr=-0.9 | corr=-0.7 | corr=-0.3 | corr=0 | corr=0.3 | corr=0.7 | corr=0.9 |
| --- | --- | --- | --- | --- | --- | --- | --- | --- |
| 1 | random | 8.995 | 8.163 | 80.732 | 6.335 | 7.917 | 12.190 | 11.440 |
| 2 | max(y) | 106.287 | 11.848 | 19.486 | 20.691 | 17.370 | 10.001 | 8.574 |
| 3 | max(x) | 1.446 | 1.297 | 0.992 | 0.776 | 0.589 | 0.412 | 0.383 |
| 4 | min(z) | 131.176 | 12.420 | 5.636 | 9.436 | 6.493 | 6.991 | 9.614 |
| 5 | max(y)max(x) | 1.554 | 1.393 | 1.003 | 0.692 | 0.469 | 0.348 | 0.340 |
| 6 | max(y)min(z) | 148.288 | 21.529 | 16.576 | 15.011 | 11.756 | 17.360 | 23.556 |
| 7 | max(x)min(z) | 1.233 | 0.838 | 0.777 | 0.766 | 0.793 | 0.838 | 1.202 |
| 8 | max(y)max(x)min(z) | 1.677 | 0.790 | 0.659 | 0.569 | 0.632 | 0.763 | 1.690 |
| 9 | lijphart | 4.187 | 2.538 | 1.642 | 1.772 | 2.002 | 2.222 | 3.240 |
| 10 | augmented lijphart | 1.199 | 0.829 | 0.778 | 0.764 | 0.790 | 0.827 | 1.106 |
| 11 | weighted max(x)min(z) | 1.129 | 0.825 | 0.792 | 0.769 | 0.753 | 0.723 | 0.876 |

Note: The table displays the root mean squared error. Smaller numbers indicate higher reliability.
